# Differences between pre-existing type and de novo type left convex thoracolumbar / lumbar scoliosis

**DOI:** 10.1186/1748-7161-10-S1-O33

**Published:** 2015-01-19

**Authors:** Takahiro Iida, Yasumasa Ohyama, Jyunya Katayanagi, Kazuyuki Matsumoto, Hirokazu Furukawa, Takashi Tomura, Satoru Ozeki

**Affiliations:** 1 Department of Orthopaedic Surgery, Dokkyo Medical University Koshigaya Hospital, Japan

## Introduction

Lenke 5C type adolescent idiopathic scoliosis (AIS) with a Cobb angle of over 30 degrees has high risk of progression. The need for corrective surgeries for degenerative lumbar scoliosis has been increasing these days and some of those cases are pre-existing type scoliosis. However, it is said to be difficult to differentiate pre-existing type scoliosis from de novo type scoliosis. The purpose of this study is to analyze the relevant X ray metrics of degenerative lumbar scoliosis and to discover differences between pre-existing and de novo type scoliosis.

## Methods

Of 54 consecutive patients who were diagnosed as candidates for corrective surgery for left convex thoracolumbar / lumbar scoliosis since December 2008, 19 patients over age 50 were included in this study. The average age was 60 years old (50-80 years old). All patients were female. Coronal and Sagittal parameters were contrasted between two groups divided according to the existence of scoliosis in their adolescence; clear (AIS) and unclear (de novo).

## Results

**Figure 1:**
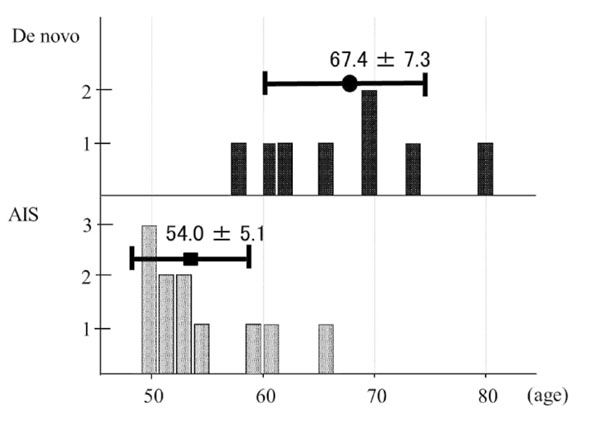
**Age distribution of both group** The average age was significantly higher for de novo than for AIS.

**Figure 2:**
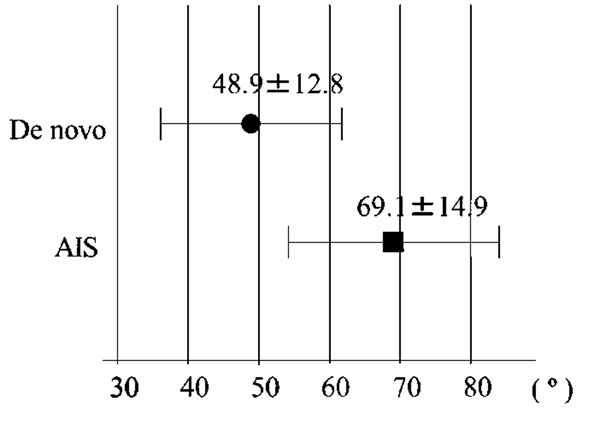
**Cobb angle** Cobb angle was found to be significantly greater in AIS.

**Figure 3:**
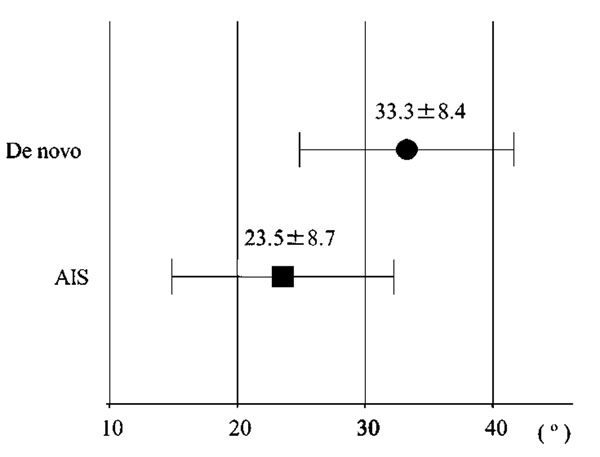
**Pelvic tilt** Pelvic tilt showed significantly larger in De novo.

Eleven were AIS, and 8 were de novo. The average age was 54.0 years old for AIS and 67.4 for de novo (p<0.05, Fig. 1). Cobb angles (69°, 49°) and L4 tilt (30°, 22°) were found to be significantly greater in AIS (Fig. 2). Nash-Moe rotation assessment showed that rotational deformity was greater in AIS type than in de novo type. Lumbar lordosis (28°, 32°), thoracolumbar kyphosis (24°, 12°), sagittal vertical axis (37mm, 58mm), and pelvic incidence (51°, 60°) showed no significant difference between the groups, however, pelvic tilt (24°, 33°) showed significant difference (Fig. 3).

## Conclusion

Among patients over 50 with degenerative thoracolumbar / lumbar scoliosis, those with pre-existing type scoliosis were found to have greater Cobb angle, greater L4 tilt, greater rotational deformity, less pelvic tilt, and were candidates for surgery at a younger age than those with de novo type scoliosis. In other words, those with de novo type scoliosis have less coronal deformity and worse sagittal pelvic alignment than those with pre-existing type scoliosis and are not considered candidates for surgery until a more advanced age. This study demonstrates some differences between pre-existing and de novo type scoliosis, contrasts the natural history of the two types of candidates for thoracolumbar / lumbar scoliosis surgery, and suggests the importance of performing surgery for Lenke 5C type adolescent idiopathic scoliosis at a younger age.

